# High or low? Comparing high and low-variability phonetic training in adult and child second language learners

**DOI:** 10.7717/peerj.3209

**Published:** 2017-05-30

**Authors:** Anastasia Giannakopoulou, Helen Brown, Meghan Clayards, Elizabeth Wonnacott

**Affiliations:** 1 School of Psychology, University of Bedfordshire, Luton, UK; 2 Department of Psychology, University of Warwick, Coventry, UK; 3 Department of Linguistics, School of Communications Sciences and Disorders, McGill University, Montreal, QC, Canada; 4 Psychology and Language Sciences, University College London, University of London, London, UK

**Keywords:** High-variability perceptual training, Child second language learning, L2 phonetic contrasts

## Abstract

**Background:**

High talker variability (i.e., multiple voices in the input) has been found effective in training nonnative phonetic contrasts in adults. A small number of studies suggest that children also benefit from high-variability phonetic training with some evidence that they show greater learning (more plasticity) than adults given matched input, although results are mixed. However, no study has directly compared the effectiveness of high versus low talker variability in children.

**Methods:**

Native Greek-speaking eight-year-olds (*N* = 52), and adults (*N* = 41) were exposed to the English /i/-/ɪ/ contrast in 10 training sessions through a computerized word-learning game. Pre- and post-training tests examined discrimination of the contrast as well as lexical learning. Participants were randomly assigned to high (four talkers) or low (one talker) variability training conditions.

**Results:**

Both age groups improved during training, and both improved more while trained with a single talker. Results of a three-interval oddity discrimination test did not show the predicted benefit of high-variability training in either age group. Instead, children showed an effect in the *reverse* direction—i.e., reliably greater improvements in discrimination following single talker training, even for untrained generalization items, although the result is qualified by (accidental) differences between participant groups at pre-test. Adults showed a numeric advantage for high-variability but were inconsistent with respect to voice and word novelty. In addition, no effect of variability was found for lexical learning. There was no evidence of greater plasticity for phonetic learning in child learners.

**Discussion:**

This paper adds to the handful of studies demonstrating that, like adults, child learners can improve their discrimination of a phonetic contrast via computerized training. There was no evidence of a benefit of training with multiple talkers, either for discrimination or word learning. The results also do not support the findings of greater plasticity in child learners found in a previous paper (Giannakopoulou, Uther & Ylinen, 2013a). We discuss these results in terms of various differences between training and test tasks used in the current work compared with previous literature.

## Introduction

### Phonetic training studies in adults

One of the most challenging aspects of learning a second language (L2) is learning to accurately perceive novel phonetic categories. This is particularly difficult when the mapping between phonetic properties and phonological categories is mismatched between the first language (L1) and L2 (Best, 1995; Flege, 1995; Giannakopoulou, Uther & Ylinen, 2011). A substantial body of literature has demonstrated that adult learners can improve their discrimination and identification of nonnative speech sounds through phonetic training, but that effective generalization may depend upon encountering sufficiently varied stimuli during training. For example, in an early attempt to train a nonnative contrast, Strange & Dittmann (1984) trained native Japanese speakers on the English /r/-/l/ contrast using a discrimination task in which participants made same–different judgments about stimuli from a synthetic *rock-lock* continuum, receiving immediate trial by trial feedback. Variability was present in the form of the ambiguous intermediate stimuli along the continuum, however, there was a single phonetic context and a single (synthesized) talker. Participants were given a variety of discrimination and identification tasks pre- and post-training. These revealed improvements in discrimination and identification for stimuli on the synthesized *rock-lock* continuum, and for novel items on a synthesized *rake-lake* continuum, but *not* for naturally produced minimal pair items that had not been encountered in training. Later experiments suggested that this limited generalization was due to the low-variability present in the stimuli used in the training intervention (i.e., a single phonetic context and single talker).

Logan, Lively & Pisoni (1991) also trained Japanese speakers on the English /r/-/l/ contrast, but used *high-variability* training stimuli that included multiple natural exemplars (67 minimal pairs, where the target speech sounds appeared in different phonetic contexts) and multiple talkers (four males and two females). They employed a minimal pair identification task in which participants identified the correct member from a (written) minimal pair, receiving trial by trial feedback. Comparison of performance on tests administered pre- and post-training revealed improvements in tasks that involved both trained stimuli *and* untrained stimuli produced by a new talker. Lively, Logan & Pisoni (1993) replicated this finding of generalization after high-variability training and, in a follow up experiment using the same training paradigm but with training stimuli produced by a single (natural) talker (although still exemplifying the contrast in multiple phonetic environments), they found improvements between pre- and post-test for the trained talker, but *not* for an untrained talker, suggesting a specific role for talker variability in high-variability training.

Following the work of Logan, Lively & Pisoni (1991), high-variability phonetic training has become a standard methodology in the field. The effectiveness of this type of training has been demonstrated for various phonetic contrasts and in studies which have also found benefits for long-term retention and in production tasks (Bradlow et al., 1997, 1999; Lively et al., 1994).

### Phonetic training studies with children

To date, studies of L2 phonetic training have primarily been conducted in adults. There is reason to predict that child L2 learners might outperform adults in these tasks, due to enhanced brain plasticity. A large body of research reports declines in language learning capacities with age (Johnson & Newport, 1989; Lenneberg, 1967; Newport, 1990, also see Kuhl, 2004, for review), with various theories proposed to account for this including changes to developing cognitive mechanisms (Newport, 1990) and increased neural commitment to structures necessary for the L1 (Kuhl, 2004). For phonetic learning specifically, there is some naturalistic support for a benefit of age in L2 speech sound discrimination coming from longitudinal studies comparing child and adult L2 learners in immersion situations. These studies show better L2 speech sound discrimination in children compared to adults (Aoyama et al., 2004; Tsukada et al., 2005), although studies comparing adults and children after periods of immersion less than one year, do not always find clear perception advantages for children (e.g., Baker et al., 2008; Snow & Hoefnagel-Höhle, 1978). However, a limitation to these naturalistic studies is that, while they control for length of residence in the L2-speaking country, the actual input received by the learners was not controlled. As Tsukada et al. (2005) point out, it is possible that children may be immersed in a more L2-rich environment than adults, making it difficult to pull apart age effects from differences in input.

Turning to training studies, only a handful of experiments have compared children and adults. These have all used high-variability phonetic training but have produced mixed results with regards to the role of age. Wang & Kuhl (2003) conducted a two-week computer-based Mandarin tone training study in which native English-speaking adults and children (three age groups: 6, 10, and 14-year-olds), with no previous experience of Mandarin, were trained to associate tones with symbols (i.e., a picture of an animal for each of the four trained tones). Training stimuli exhibited high-variability (648 stimuli produced by six native speakers of Mandarin). Overall, older participants outperformed younger participants (adults >14 years >10 years >6 years) at both the pre- and post-training tests. However, although all participant groups improved as a result of training, the amount of improvement was approximately the same across all age groups. This result thus does not support an account in which younger learners demonstrate greater plasticity.

Wang & Kuhl (2003) suggest that one possible explanation for the fact that they did not see plasticity differences for children and adults is that, for tones, English speakers do not have pre-existing comparable categories. This means that the “mental map” for tone is equally open for children and adults, so that age effects due to previous L1 experience are not expected. This would predict that plasticity effects should be more likely in segmental phonology. However, a similar result was found by Heeren & Schouten (2008, 2010), who trained adults and 12-year-old native Dutch speakers, with no previous Finnish experience, to discriminate the Finnish length contrast /t/-/t:/. This was a five-day study with a pre-test and post-test on the first and last days and three days of training. Training consisted of an identification paradigm (participants identified stimuli as “short t” or “long t” and received feedback). Stimuli were seven-step continua created from recordings of five talkers. Pre- and post-tests included identification and discrimination within and across category boundaries. Although adults again out performed children overall, both age groups showed reliable increases in sensitivity in the newly trained category boundaries and, critically, there were again similar levels of improvement in both age groups. This result might appear to corroborate that of Wang & Kuhl (2003), however, the lack of age effects may be due to a different reason, specifically, the amount of training provided (three training sessions) may be insufficient in order for children to outperform adults (c.f., Giannakopoulou, Uther & Ylinen, 2013a, discussed below).

In contrast to the studies discussed above, two further studies *have* found evidence of greater learning in children than adults for high-variability phonetic training. Shinohara & Iverson (2013, 2015; also Shinohara, 2014) compared learning of the English /r/-/l/ contrast in native Japanese adults (25–59 years), adolescents (15–18 years) and older and younger children (8–12 and 6–8 years). Training stimuli exhibited high-variability (100 word-initial minimal pairs from five talkers) and involved ten days of minimal pair identification (with written stimuli) and discrimination tasks (all with feedback). There were pre- and post-test identification tasks with new talkers in both trained (word-initial) position and untrained positions. In both identification accuracy and category discrimination abilities, all groups showed evidence of learning and generalization to new speakers and phonetic contexts. However, adolescents and older children improved more than either 6–8 year-olds or adults. Shinohara & Iverson (2013, 2015) interpret the increased learning in older children and adolescents compared with adults as being due to their less fossilized brain plasticity and lesser interference from developed L1 phonetic units. Lesser learning in the 6–8 year-olds, which was unpredicted in a plasticity account, was explained as being a result of difficulties with the tasks due to an immaturity of phonemic awareness.

One difference between the paradigms used by Heeren & Schouten (2008, 2010) and Shinohara & Iverson (2013, 2015) is the length of training: Heeren & Schouten used three training sessions whilst Shinohara & Iverson used 10. If children’s early learning is slower than that of adults (Snow & Hoefnagel-Höhle, 1978), this could potentially account for why Shinohara & Iverson saw a plasticity benefit (at least for older children compared to adults), whilst Heeren & Schouten did not. Some evidence for this comes from a final study by Giannakopoulou, Uther & Ylinen (2013a), who also found a plasticity benefit (greater learning in 7–8 year-olds than in adults), but also found evidence that this maturational difference only emerged after several sessions of exposure. This study used high-variability phonetic training to train the tense-lax English vowel contrast /i/ versus /ɪ/ (e.g., *bean-bin*) with child (7–8 years) and adult (20–30 years) native Greek learners of L2 English. The study explored both age effects and cue weightings, through the use of natural and modified duration stimuli (whereby duration cues were equalized and not relevant, or were reliable cues). A pre-test, training, post-test paradigm was used, with training consisting of 10 sessions using an identification task (identifying the correct member of a minimal pair given written stimuli) with high-variability stimuli (45 minimal pairs produced by two male and two female speakers). Half of the participants in each age group were trained with modified stimuli (no duration cues) and half with natural stimuli. Participants were given the option to replay any given stimulus and feedback was provided in the form of a video game style animation. Although Greek adults, who started with more years of L2 education, generally performed better than Greek children at pre-test, high-variability perceptual training improved performance for both groups (child and adult) across all tasks (perceptual identification and discrimination for both natural and modified stimuli conditions). However, critically, children improved more than adults. Importantly, the results from Giannakopoulou, Uther & Ylinen (2013a)’s training task, which were recorded each day, suggested that children’s identification performance only overtook that of adults by Session 7 (see Giannakopoulou, Uther & Ylinen, 2013a, Figs. 11–14). This suggests that plasticity benefits, which are seen in this study and in that of Shinohara & Iverson, but not in Heeren & Schouten (2008, 2010) studies could rely on more lengthy exposure. However, this cannot account for why Giannakopoulou, Uther & Ylinen saw this benefit for 7–8 year-olds compared with adults while this was *not* seen for 6–8 year-olds compared with adults in the Shinohara & Iverson study (only for older children compared with adults). If Shinohara & Iverson are right that their youngest children had greater difficulty with the learning task due to less well developed phonemic awareness, one possibility is that the /i/ versus /ɪ/ contrast is somehow more salient for Greek speakers than the /l/ versus /r/ contrast is for Japanese speakers, even for younger children. This may be partially due to the length cue present in these stimuli: the difference between children and adults was more marked for the natural stimuli compared with the modified stimuli, suggesting that the children may have been particularly relying on durational cues during training. This is in line with research showing that nonnative listeners from many different language backgrounds tend to rely heavily on the duration cue when discriminating English tense-lax vowel pairs (unlike native listeners who rely more on formant frequency, e.g., Flege, Bohn & Jang, 1997). The reliance on durational cues may also have been exacerbated by the use of written stimuli during training since English spelling provides an additional analogue length cue (two letters such as *ee* and *ea* are often used to represent the longer vowel /i/ while a single letter such as *i* is more often used to represent the shorter vowel /ɪ/), which may aid learning (see also Giannakopoulou, Uther & Ylinen, 2013b, for evidence on the role of orthography in learning this contrast). We return to this point in the General Discussion.

In sum, the results of a handful of phonetic training studies give mixed results with respect to whether younger learners show stronger learning given matched input (i.e., plasticity effects). It has been suggested that that the presence (or lack) of pre-existing comparable categories in the L1, the length of exposure, and the salience of the contrast could all influence children’s learning and contribute to differences across experiments. In the current study, we build on the paradigm established in Giannakopoulou, Uther & Ylinen (2013a)—where plasticity benefits were clearly seen—comparing Greek 7–8 year-olds and adults trained on the English /i/ versus /ɪ/ contrast (though we modify the paradigm to remove orthographic cues to learning). We use this paradigm to explore the effect of variability in training with different age groups.

### Comparison of high and low-variability training

As discussed above, work by Lively, Logan & Pisoni (1993) suggested a benefit for high-variability input over low-variability input in phonetic training for adult learners. The finding that encountering varied training instances boosts generalization is intuitively sensible: experience of variation allows the formation of generalized representations that include only phonetically relevant cues and exclude irrelevant talker identity cues. Since the seminal experiments of Lively, Logan & Pisoni (1993), many phonetic training studies have continued to use high-variability input, however, surprisingly few have actually tested the benefit of high-variability directly. One study which did find a high-variability benefit was conducted by Clopper & Pisoni (2004), although this focused on dialect categorization rather than L2 phonetic learning. They tested participants’ ability to categorize dialects following exposure to high-variability training (three talkers per dialect) compared with low-variability training (one talker per dialect), finding better generalization after high-variability training.

However, two studies looking at lexical tone learning also compared variable (multispeaker) training with less variable training, but did *not* find an overall benefit of high-variability input (Peracchione et al., 2007; Sadakata & McQueen, 2014). Instead, these studies found an interaction with aptitude (i.e., baseline perceptual ability for detecting pitch) whereby only high aptitude participants benefitted from high-variability input whilst those of low aptitude did better with low-variability training. However, since these studies did not use segmental phonetic contrasts it is currently unclear the extent to which they would generalize to phonetic training. Finally, one recent training study did specifically compare high- and low-variability in L2 phonetic learning at the segmental level: Sadakata & McQueen (2013), tested native Dutch speakers’ ability to identify geminate and singleton variants of the Japanese fricative /s/, comparing low and high-variability training (here manipulated in terms of item variability). They found that more variable input did lead to greater improvements with generalization but no interaction with aptitude.

To the best of our knowledge the studies reviewed above are the only ones to directly compare low- and high-variability training for phonetic contrasts. Moving beyond the phonetic learning literature, a separate literature on L2 word learning has also explored the benefits of input variability (Barcroft & Sommers, 2005, 2014; Sommers & Barcroft, 2007, 2011). A general finding from this literature is that vocabulary learning, as tested both in production and reception tests, is stronger when exposure is more varied, with benefits both of multiple talkers and multiple voice quality types. Further experiments rule out explanations in terms of a benefit of greater cognitive effort (Sommers & Barcroft, 2011). Barcroft & Sommers (2005, 2014) explain this general benefit of acoustic variability in terms of an exemplar-based framework whereby indexical information from all encountered examples is retained in the early stages. This means that when words are encountered from multiple talkers/voice types, learners incorporate a wider variety of cues into their representations, allowing them to form more “associative hooks” and robust representations for the target words. Note that this explanation is subtly different from the standard explanation as to why high-variability input benefits phonetic learning, which is assumed to stem from learning to ignore phonemically irrelevant information and thus is specifically important for generalization (whereas the benefit for word-learning tasks should hold for both trained and novel talkers at test).

Turning to children, no study has specifically explored whether high-variability input is more effective than low-variability input for L2 phonetic training in children. However, there is some research into word learning in L1 which suggests a role for high-variability in infants. This research has been conducted with infants in the early stages of word learning (around 14 months). A surprising finding with this age group is that even if they have mastered a particular L1 phoneme contrast (i.e., they discriminate between relevant phoneme contrasts and fail to discriminate nonnative contrasts) they may have difficulties learning new words that differ by this contrast. For example, Stager & Werker (1997) found that when 14-month-olds were exposed to two novel words which formed a minimal pair (/bɪ/ and /dɪ/) paired with two novel objects, they did not later differentiate between trials in which the word-object pairing was identical versus opposite to that previously seen in habituation (the so-called “switch task”). This is despite the fact that children of this age were shown to be able to discriminate /b/ and/d/ outside of the context of a word-learning task. This effect has been demonstrated many times (see Werker & Curtin, 2005, for a review), critically, however, Rost & McMurray (2009) demonstrated that it is affected by the variability of the exposure set. Using a similar switch task to Stager & Werker (1997), they replicated the null effect when the novel words (/buk/ and /puk/ in their study) were produced by a single talker, but showed that infants of the same age *did* differentiate between the minimal-pair novel words when exposed to the novel words spoken by multiple talkers. Rost & McMurray (2010) further probed what aspect of the variability in the input was beneficial for word learning. They considered the possibility that, although infants could discriminate the phonetic contrast in question (/b/-/p/) their phonetic categories might still be developing. In this case, the critical aspect of variability might be the presence of a bimodal distribution of the most relevant cue for differentiating the contrast (see also Maye, Werker & Gerken, 2002—in this case voice onset time (VOT)). Rost & McMurray (2010) tested this by varying VOT in a clear bimodal distribution while holding talker constant. Surprisingly, infants were *not* able to differentiate the minimal pair after exposure to these stimuli, nor were they able to do so when exposed to the items spoken by a single talker but with variation in multiple acoustic cues to the contrast (VOT, F0 transition, and burst amplitude). In contrast, infants *were* able to discriminate between the minimal pairs when exposed to the items spoken by multiple talkers but with a fixed VOT across talkers. This unexpected finding suggests that what is critical is variability arising from talker differences but on *irrelevant* dimensions to the contrast in question. Apfelbaum & McMurray (2011) provide an account of this in terms of associative learning. Via computational simulation, they show that the experimental results are predicted if word learning involves associating all available acoustic cues with objects, including both relevant (e.g., place of articulation/voicing) and irrelevant (e.g., talker) cues. The intuition behind the model is that associative learning will pick up on any *consistent* relationships across instances, meaning that both relevant and irrelevant acoustic cues will become associated with an object if they are highly consistent, as is the case when tokens all come from the same talker and thus are highly similar. The association of these irrelevant talker cues reduces the contrast established by the phonetic cues since they are shared across the words and provide evidence that they are the same. Note that a similarity between this explanation and that of Barcroft & Sommers (2005, 2014) is that they both assume that irrelevant cues are initially incorporated into lexical representations. There is some independent evidence of this in L1 learning (Singh, Morgan & White, 2004; Singh, White & Morgan, 2008).

In sum, there is a long-standing assumption that more variable input is more beneficial in L2 phonetic training, although very few published studies have actually directly tested this in adults, and none have done so in children. There is also evidence that variable input is beneficial in adult L2 and infant L1 vocabulary learning, which has been interpreted in terms of the formation of robust, speaker independent representations.

### Current study

The current experiment adds to the small literature exploring phonetic training in both adults and children. We build on the work of Giannakopoulou, Uther & Ylinen (2013a) and focus on learning of the nonnative English /i/-/ɪ/ contrast by adult and 7–8 year-old native Greek speakers. Our central research question was whether variability would benefit learning. Although variability can be manipulated along a number of dimensions, we chose to manipulate talker variability since this type of variability has been explored across both the phonetic and word learning literatures. To this end we compared learning from high-variability (four talkers) versus low-variability (one talker) input, with overall exposure matched across conditions. We embedded the phonetic training task in a word-learning task, whereby training involved pairing minimal pairs with picture representations of the two words (e.g., hear *sheep*, choose between pictures of a *sheep* and a *ship*), conducted in a child friendly computerized training environment. This choice allowed us to investigate the effect of variability on learning at both the phonetic and lexical levels. In addition, using pictures allowed us to avoid using orthography in training, addressing the concern that learners in Giannakopoulou, Uther & Ylinen (2013a) may have relied on length cues in English spelling (e.g., *sheep versus ship*) as an aid to learning. We matched other aspects of the training to that in Giannakopoulou, Uther & Ylinen (2013a), to allow reasonably direct comparison between the studies. For example the number and duration of the training sessions were approximately the same, training used an animation to provide feedback and a “replay” button allowed participants to repeat stimuli if necessary.

However, our test of phonetic learning was a three-interval oddity discrimination task, rather than an identification task, in order to avoid using orthography but still be able to test both trained and untrained items. The inclusion of untrained items was important since high-variability is specifically predicted to benefit generalization—i.e., exposure to multiple talkers should aid the ability to ignore phonetically irrelevant information. Voice novelty and word novelty were manipulated separately (since it is possible that exposure to multiple speakers might specifically benefit generalization across talkers, rather than generalization more broadly).

Our primary measure was the extent to which training strengthened lexical representations, since we assumed that our participants would begin the study with some knowledge of the words. We chose to focus on the links between the forms and their meanings and used a primed auditory lexical decision task to tap semantic representations in the L2 via cross-language priming (i.e., semantic priming from L2 to L1). This was adapted from a task previously used to test vocabulary development in an artificial language learning experiment (Tamminen & Gaskell, 2012). Comparison of pre- and post-training performance tested whether this aspect of participants’ representations was changed by the training process, and whether the extent of change was modulated by input variability. We also included a more direct measure of vocabulary learning at both pre- and post-test. Participants heard Greek L1 words and matched these to the correct translation from one of four English L2 words, all taken from the training set (no minimal pairs included). This task served multiple purposes. First, it allowed us to determine which words a participant was familiar with prior to the study. Second, it allowed us to measure vocabulary learning between pre-and post-test, and finally, it ensured that all participants began the training task with some knowledge of the word meanings.

Our main prediction was that, for both measures of phonetic learning and word learning, there should be greater improvements from pre- to post-test for the high-variability condition compared to the low-variability condition. This was based both on the theoretical benefits of boosting generalization discussed above, and on the existing empirical literature (although as has been seen, there are some substantial gaps in this literature, particularly for phonetic learning and particularly in children).

A second prediction following Giannakopoulou, Uther & Ylinen (2013a) was that in the phonetic learning tasks (i.e., in the training and three-interval oddity discrimination tasks), children would show stronger learning effects than adults.

## Materials and Methods

### Ethics statement

This project received ethical consent by the ethical committee of the University of Warwick (Ethical Application Ref: 80/12-13), abiding to the ethical standards of the Declaration of Helsinki. For children, written informed consent was obtained from their parents prior to the first session. Adults provided written consent at the beginning of the first session. Participants received a certificate and small gift at the end of the experiment.

### Participants

Child (*M* = 8.4; SD = 0.6; range = 7;8 – 9;8 years) and adult (*M* = 24.3; SD = 4.3; range = 18;3 – 32;3 years) native Greek-speaking learners of English were recruited from primary schools and higher education colleges in Athens. Participants were tested in school/college by researchers or class teachers who were provided with instructions for running the experiment. The final sample consisted of 52 children and 41 adults. Participants were randomly assigned to each of two counterbalanced versions of the two experimental conditions (high-variability version1/version2 versus low-variability version1/version2). Although we had planned for participants to be spread equally across conditions and versions, our final sample was uneven. Additional participants were tested but we were unable to use their datasets due to their dropping out of the experiment, recording errors, and other errors in testing due to difficulties of testing on multiple computers in a busy school environment, and that some of our testing was done by school staff. We retained participant’s datasets where there was data for the discrimination and lexical decision tasks (pre- and post-test), and where at least 60% of their training data had been recorded. Note that statistical analyses which allow for an uneven balance across conditions and versions were used. Details of participants in each condition are given in Table 1.

**Table 1:** Participant details.

			*N*	Gender	Mean age	SD age
Adults	High-variability	(Version 1)	11	2M, 9F	25:1	5:0
	High-variability	(Version 2)	11	3M, 8F	24:4	3:1
	Low-variability	(Version 1)	10	1M, 9F	24:9	3:9
	Low-variability	(Version 2)	9	4M, 5F	22:8	5:1
Children	High-variability	(Version 1)	14	7M, 7F	8:7	0:7
	High-variability	(Version 2)	14	7M, 7F	8:10	0:5
	Low-variability	(Version 1)	11	6M, 5F	8:10	0:6
	Low-variability	(Version 2)	13	5M, 8F	8:8	0:5

Participants lived in Greece and were students of L2 English. All participants had normal or corrected-to-normal vision and nonimpaired hearing, and none reported having a language or learning disorder. Child participants’ level of proficiency was basic (L2 English education, *M* = 1.96 years, range = 1–2 years). Adult participants’ level of proficiency was advanced (L2 English education, *M* = 10.98 years, range = 9–13 years). All participants had spent less than two weeks in an English-speaking environment.

### Stimuli

The experimental stimuli consisted of 20 real-word minimal pairs (e.g., *ship-sheep*) and 20 nonword minimal pairs (e.g., *stin-steen*) containing the English tense-lax vowel distinction (nonword minimal pairs were created so that they matched the real-word minimal pairs as closely as possible in their final syllables; see Table S1 for a list of all stimuli). Participants learned the real-word minimal pairs in the training task, but were tested on both these real-word items from training and nonword minimal pairs not included in training. This allowed us to test both trained and novel items.

All minimal pairs were recorded by five native English speakers (three female, two male) with Southern British English pronunciation using a micro track 24/96 digital recorder. Words were edited into separate sound files, and peak amplitude was normalized using Audacity® (2012). All other natural variation between recordings was kept. Clipart pictures of the 40 English words were selected from free online databases.

In addition to the main experimental stimuli, a second set of stimuli were developed for a primed auditory lexical decision task. In this task, primes could be either English words or Greek words, and targets were either semantically related to the prime, semantically unrelated to the prime or nonwords (see Table 2 for example and Table S2 for a full list of these stimuli).

**Table 2:** Examples of the different trial types in the primed auditory lexical decision task.

Trial type	English prime	Greek prime
Semantically related target	*sheep—πρόβατο (sheep)*	*σκύλος (dog)—γάτα (cat)*
Semantically unrelated target	*sheep—γόνατο (knee)*	*σκύλος (dog)—δέμα (parcel)*
Nonword target	*sheep—γραμόλι*	*σκύλος (dog)—δούμα*

For the English primes, one word from each real-word minimal pair was selected (12 /i/ items, eight /ɪ/ items). Originally, we had selected 10 /i:/ and 10 /ɪ/ items, but as two of the /ɪ/ items had Greek translations that were phonologically very similar to the English word (gin, dip) these items were replaced with their /i:/ counterpart (gene, deep) since the primed auditory lexical decision task aimed to examine semantic (not phonological) priming. For the Greek primes, 20 words that matched the English primes as closely as possible in frequency were selected. English word frequency was taken from the MRC Psycholinguistic Database (Wilson, 1988). Greek word frequency was obtained from the GreekLex database (Ktori, van Heuven & Pitchford, 2008). An independent-samples *t*-test confirmed that the two lists of prime words did not differ significantly in frequency, *t*(38) = −1.12, *p* = .27. In addition, the number of nouns, verbs, and adjectives were identical in both lists.

The semantically related target word for the English primes was the Greek translation. For the Greek primes, the semantically related word was selected by asking 15 native Greek speakers to write down the first word that came to mind for each Greek prime word. For both types of prime word, semantically unrelated words were words which were unrelated in meaning with the target words which were matched as closely as possible in frequency and length to the semantically related words (frequency, *t*(78) = −0.13, *p* = 0.90; N phonemes, *t*(78) = 0.13, *p* = 0.89; N syllables, *t*(78) = 0.00, *p* = 1.0). Nonwords targets for both English and Greek prime words were generated by changing one and two syllables of real Greek words, preserving the number of consonants and vowels, and were matched as closely as possible in length to the word targets (N phonemes, *t*(158) = 0.98, *p* = 0.33; N syllables, *t*(158) = 0.99, *p* = 0.33). Primes and targets were never cohorts or rhymes.

All Greek word and nonword stimuli for the lexical decision task were recorded by a native Greek speaker, and were edited as above.

### Design

**Figure 1:**
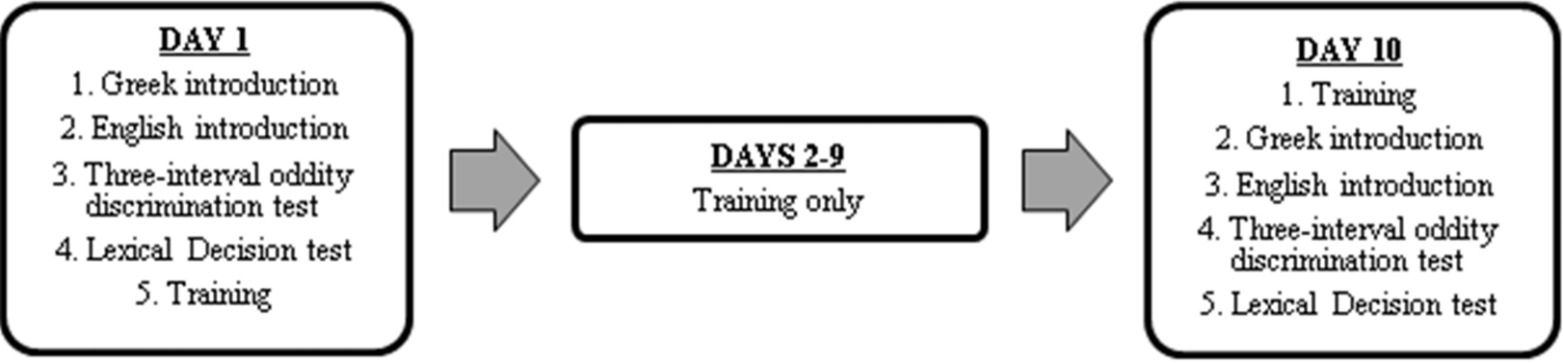
Tasks completed in each of the 10 experimental sessions.

Each participant completed 10 sessions over approximately two weeks (one session per day over a minimum of 12 and maximum of 15 days). The experiment involved three stages: pre-test, training, and post-test. The pre- and post-tests were identical, and contained five tasks (see Fig. 1). Session 1 began with the pre-test, followed by a block of training. Sessions 2–9 consisted of training only. Session 10 consisted of training, followed by the post-test.

There were two experimental conditions that differed only during training—high-variability versus low-variability. In the high-variability training, English minimal-pair words were spoken by four different talkers (two female, two male). In the low-variability training, English minimal-pair words were spoken by a single talker (always female). Pre- and post-tests were identical for the two conditions (although old/new talker was counterbalanced across participants in the discrimination task, resulting in four counterbalancing conditions; Table 3).

**Table 3:** Counterbalancing of the English talkers in each task.

Task	High-variability 1	High-variability 2	Low-variability 1	Low-variability 2
English introduction	Female 1	Female 2	Female 1	Female 2
Three-interval oddity discrimination	Female 1 (old)	Female 1 (new)	Female 1 (old)	Female 1 (new)
	Female 2 (new)	Female 2 (old)	Female 2 (new)	Female 2 (old)
Primed auditory lexical decision	Female 1	Female 2	Female 1	Female 2
Training	Female 1	Female 2	Female 1	Female 2
	Female 3	Female 3		
	Male 1	Male 1		
	Male 2	Male 2		

### Procedure

All tasks were run using Exbuilder (a custom built software package developed at the University of Rochester) on laptop or desktop computers in quiet classrooms. Multiple participants were tested simultaneously on separate computers. Stimuli were presented binaurally over headphones at a comfortable listening level.

#### Greek introduction

**Figure 2:**
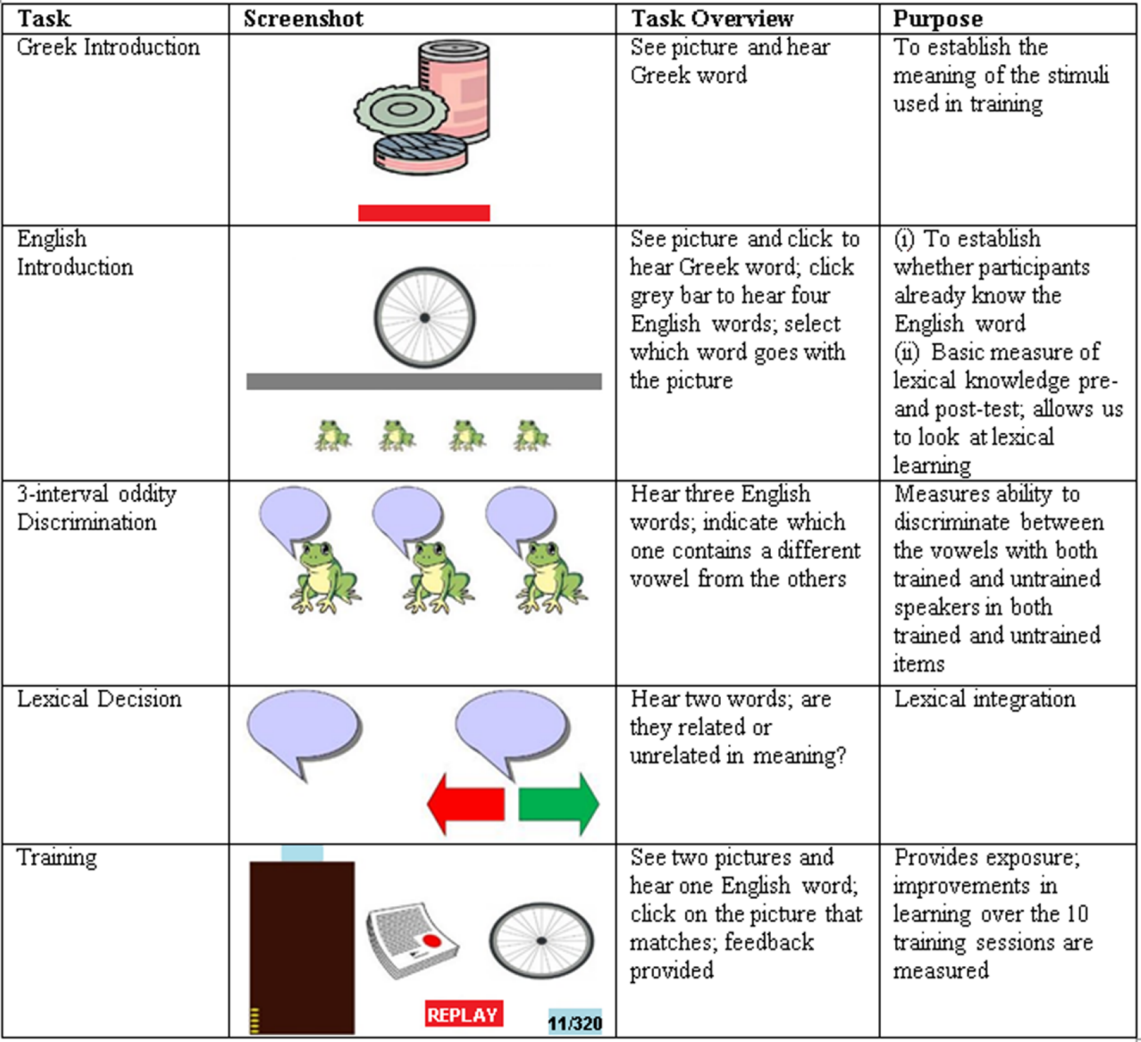
Overview of the five experimental tasks.

In this task, a picture of one of the minimal-pair words was presented centrally on the screen, and participants heard the corresponding Greek word (see Fig. 2). Each minimal-pair word was presented once each in a random order. This task was included to ensure that participants accessed the correct meaning for each picture since not all items were concrete nouns (e.g., *leap, slip,* etc.). No data were recorded from this task.

#### English introduction

In this task, participants saw a picture of one of the minimal-pair words, presented centrally at the top of the screen. Participants could click on this picture to hear the corresponding Greek word if required. Participants subsequently clicked on another button in the middle of the screen to hear four possible English words which were each “spoken” by one of four frogs which appeared at the bottom of the screen (see Fig. 2). If the participant selected the correct English word, they received positive feedback [Greek translation of *correct* (“*σωστό*”)] and the English word was replayed. If the wrong word was selected participants listened to the four options again and had another go. This continued until the correct word was chosen. The three distracter words for each trial were randomly selected (but identical for all participants) with the following constraints; (a) no minimal pairs were heard together (e.g., if *ship* was the target, *sheep* was not a distracter); (b) no rhyming items were heard together (e.g., if *ship* was the target, *chip* was not a distracter); (c) each word was heard once as a target, and three times as a distracter. Trials were presented in a random order. Accuracy on the first attempt was recorded. Accuracy in Session 1 provided a baseline measure of English vocabulary knowledge, whilst the change in accuracy between Sessions 1 and 10 provided a measure of vocabulary learning.

#### Three-interval oddity discrimination test

In this test, participants heard three words (played with ISIs of 200 ms) spoken by a single talker. Two words were different tokens of the same word (e.g., *sheep, sheep*), and one the other minimal pair item (e.g., *ship*). Each word was “spoken” by one of three frogs which appeared on the screen and participants clicked on a frog to indicate which word was the odd one out (see Fig. 2). A response could not be made until the third sound file had finished playing. Instructions emphasized accuracy and no feedback was provided. All 20 real word and 20 nonword minimal pairs were heard once each. Half of the trials contained an /ɪ/ target (e.g., *sheep, sheep,* ***ship***), and half contained an /i/ target (e.g., *chip, chip,* ***cheap***). To minimize the influence of duration cues (i.e., /i/ is a longer vowel than /ɪ/, so *sheep* is likely to have a longer acoustic duration than *ship*) all sound files were normalized in length by adding silence at the end, up to the length of the longest item. Thus, all trials were matched in length from the onset of the first sound file until the moment when participants could respond.

Nonword trials tested whether participants could generalize their training to new untrained items. We also tested whether participants could generalize to a new talker. To do this, 10 of the 20 real-word minimal pairs were presented in a familiar voice, and the remaining 10 in a new voice, and likewise for the 20 nonword minimal pairs. The two talkers used as the familiar/new voices were counterbalanced across participants (Table 3).

#### Primed auditory lexical decision test

A primed auditory lexical decision task investigated the status of the semantic representations for English words and determined whether this was altered following training. On each trial participants heard two words (a prime and a target). The prime could be a trained English word or a Greek word. Each prime was repeated four times, once with a semantically related word target, once with a semantically unrelated word target, and twice with a nonword target. If the trained English words have become integrated with Greek lexical knowledge then faster response times should be observed when the English prime is followed by a semantically related, compared to semantically unrelated Greek word. The inclusion of Greek primes enabled comparison of the magnitude of semantic priming effects between English and Greek. Although priming studies do not typically repeat the primes (more commonly repeating the target words with different types of primes), using the English words as primes four times in the current study increased the number of observations and thus the statistical power without the need to train participants on a very large number of English words (see Tamminen & Gaskell, 2012, for a similar design using an artificial language). The target word was unique on each trial. Examples of each trial type are provided in Table 2 and a screenshot of the task is provided in Fig. 2.

The task began with eight practice trials with feedback, followed by 160 experimental trials without feedback. Participants were instructed to make a word/nonword judgment for the second word as quickly as possible. Responses were made using the left (nonword) and right (word) arrows on the computer keyboard. Response times were measured from the onset of the second word.

#### Training

On each trial participants heard an English word and selected one of two pictures (from the same minimal pair) displayed on the computer screen (see Fig. 2). Following Giannakopoulou, Uther & Ylinen (2013a), participants could replay the English word an unlimited amount of times before making a decision. If the correct picture was selected, the incorrect picture disappeared, the English word was replayed, and a short video of a “happy” bunny jumping up and down was played. A picture of a coin also appeared in a box on the left-hand side of the screen, with the aim of motivating participants to try to earn more coins during each subsequent training session. If the incorrect picture was identified both pictures were removed from the screen and a short video of a “sad” bunny was played. The two pictures then reappeared and the English word was played again. Once the participant clicked on the correct picture feedback was provided as in correct trials but no coin was awarded. A training block consisted of the 40 English minimal-pair words each heard eight times, resulting in 320 trials, presented in a random order. In the low-variability condition, all items were spoken by the same talker. In the high-variability condition participants heard each word spoken twice by each of the four talkers (two female, two male). We aimed that participants would undertake one training block (320 trials) in each of their 10 sessions, however, due to time constraints associated with testing in schools, some children were only able to complete 180 training trials in Sessions 1 and 10. In these cases, children completed the remaining 180 trials (to make a full training block of 320 trials) in Sessions 2 and 9, respectively. Data were coded such that the first 320 trials and last 320 trials completed were coded as Sessions 1 and 10 training blocks, respectively.

## Results

### Analyses and statistical approach

Data were primarily analyzed using linear mixed effects models (Baayen, Davidson & Bates, 2008; Jaeger, 2008; Quené & van den Bergh, 2008) using the package *lme4* (Bates, Maechler & Bolker, 2013) for the R computing environment (R Core Team, 2016). Since adults and children generally had very different starting points at pre-test, the data from each age group were analyzed separately for each task. However, since we were specifically interested in age differences for phonetic discrimination, we also included additional analyses comparing the age groups for the training and three-interval oddity discrimination tasks.

Linear mixed effects models allow binary data to be analyzed with logistic models rather than as proportions, as recommended by Jaeger (2008). Our approach was to automatically include all the relevant experimentally manipulated variables for each task, and all the interactions between those variables, as fixed factors in a model, regardless of whether they contributed significantly to the model (i.e., we did not use stepwise model comparison). Since preliminary analysis suggested that the extent to which children had used the “replay” button during training was positively correlated with their increase in performance from pre- to post-test in the three-interval oddity discrimination task (*r* = 0.38, *df* = 0.91, *p* < 0.01), we also included each participant’s *mean-replay-usage* as a fixed factor in the models for that task (note that although the correlation did not hold for adults (*r* = 0.17, *df* = 0.39, *p* = 0.27), the factor was nevertheless included in both models for consistency). In addition, preliminary analyses revealed that one of the two talkers used in the test stimuli (i.e., as the trained/untrained voice; see Table 3) was more intelligible than the other, affecting discrimination. In order to ensure that key effects were not carried by a specific *talker,* we included both *talker* and all the interactions with *talker* as a fixed factor.^1^1There was just one model reported in the text where it was not possible to include the interactions with the control variable due to nonconvergence: the model predicting children’s training data using *training-session* as a continuous predictor. However, equivalent interactions were included in the follow up model which is also reported, where the training-session variable was replaced by the binary predictor *test-half.* Finally, in all models, predicting variables (including discrete factor codings) were centered to reduce the effects of collinearity between main effects and interactions, and in order that main effects were evaluated as the average effects over all levels of the other predictors (rather than at a specified reference level for each factor). We do not report full statistical models. For the experimental factors, we report statistics for main effects and interactions where there are predictions. For example, we did not inspect the model for a main effect of *voice-novelty* (trained versus untrained talker), since this effect is not interpretable for both levels of *test-session* (i.e., novelty is only relevant after training). We also inspected each model to see if there was a main effect of the control variable *talker,* and in addition, wherever we found a reliable main effect or interaction for the experimental factors we looked to see if it was qualified by a higher-level interaction with *talker.* For clarity of exposition, the results with the control variable are *not* reported in the main text (see Table S3), with the exception of places where we found a reliable interaction between an effect of interest and the control variable *talker,* and this broke down to suggest that there was an effect only for one of the talkers.

The lme4 package provided *p*-values automatically for logistic mixed effects models but not for linear mixed effect models. For models with a continuous outcome variable (i.e., RTs in the lexical decision task) *p*-values were calculated using the lmerTest package using Kenward–Roger approximation for denominator degrees of freedom. We included *participant* as a random effect and used a full random slope structure (i.e., by-subject slopes for all within-subject factors (although not the control variables) and their interactions, as recommended by Barr et al. (2013)). In some cases, the full model did not converge and here we removed the correlations between slopes (Barr et al., 2013). All of the models reported converged with bound optimization by quadratic approximation (BOBYQA optimization; Powell, 2009). The analyses scripts and output can be viewed here: http://rpubs.com/ewonnacott/247911. Data files and scripts are also available on the Open Science Framework: https://osf.io/8anzk/.

### Training

**Figure 3:**
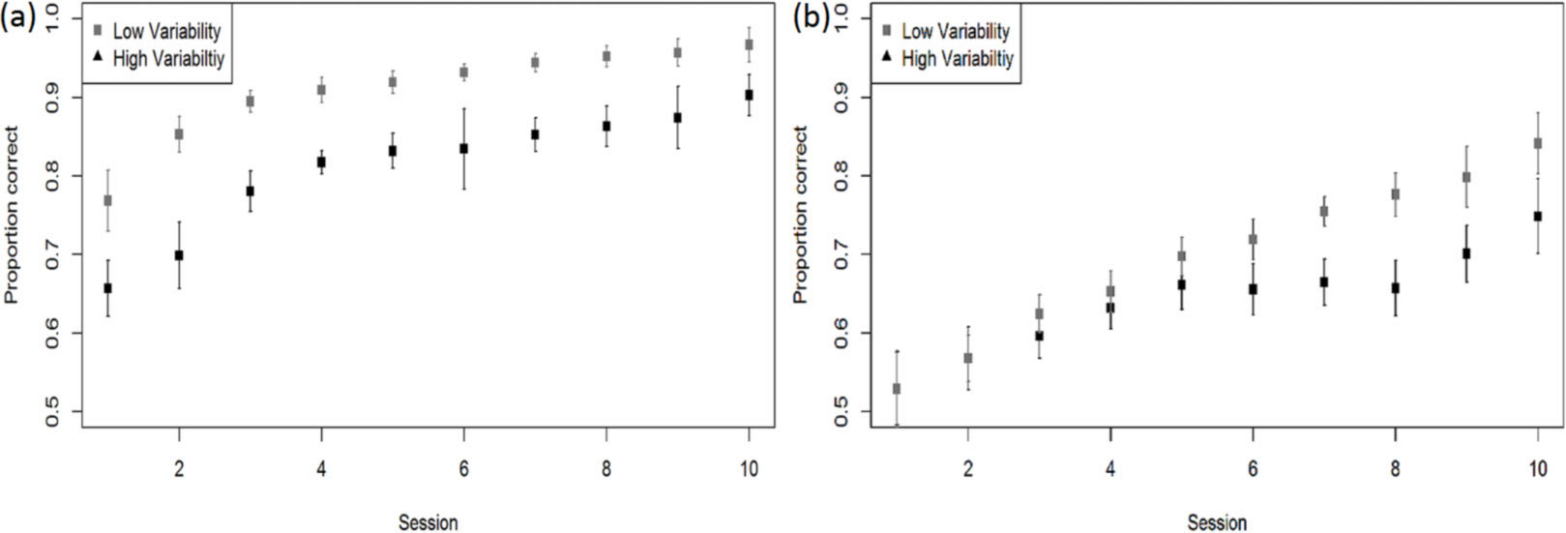
(A) Adult and (B) child performance during training (error bars show standard error). For the high-variability condition, trials with the three additional talkers are excluded (note: for all of the plots within this paper, means are corrected to control for imbalance in counterbalancing of talkers).

Participants’ accuracy in selecting the correct picture from its minimal pair on the first attempt was recorded, though with some data loss (adults = 2%; children = 6%). Recall that high-variability training comprised four talkers; for half of the participants in this condition, these were talkers Female 1, Female 3, Male 1, and Male 2, for the other participants these were talkers Female 2, Female 3, Male 1, and Male 2. In the low-variability training, half of the participants were trained with Female 1 only and half with Female 2 only (where Female 1/Female 2 were not used in training, they were used as the novel voice in testing—see Table 3). This design meant that high-variability included three talkers that were never included in the low-variability training. It is possible that stimuli produced by these talkers could be easier or harder to identify than stimuli produced by the two talkers used in both training conditions. To ensure a fair comparison across conditions, we only consider trials in the *high-variability* condition where the stimuli were produced by one of the two talkers who were also used in the *low-variability* condition (i.e., trials with Female 3, Male 1, and Male 2 were excluded; see Table 3). The proportion of correct responses in each session is shown in Fig. 3. For our primary analyses, the data were analyzed in two logistic mixed effects models predicting whether a correct response was given (1/0) on each trial. Experimental factors in the model were *training-session* (1→10) and *condition* (high-variability, low-variability), and the interaction between them. We were also interested in the contrast between age-groups, however, as can be seen in Fig. 3, by the final session, adult participants were at ceiling in the low-variability condition. We, therefore, restricted our analyses comparing age-groups to data from the adults and children in the high-variability condition only. Here, we used a logistic mixed effects model predicting response accuracy with fixed effects of *training-session, age-group* and *talker,* and all of the interactions between them, although we only report the effect of age and interactions with age.

#### Adults

There were main effects of *training-session* (*β* = 0.35, SE = 0.03, *z* = 11.21, *p* < 0.001) and *condition* (*β* = 1.63, SE = 0.25, *z* = 6.41, *p* < 0.001) and a reliable interaction between *condition* and *training-session* (*β* = 0.21, SE = 0.05, *z* = 3.86, *p* < 0.001). This reflects improvement across sessions and an overall better performance in the low-variability condition which increases with training.

#### Children

There was a reliable main effect of *training-session* (*β* = 0.22, SE = 0.03, *z* = 7.06, *p* < 0.001), reflecting improved performance across sessions, but no reliable main effect of *condition* (*β* = 0.37, SE = 0.24, *z* = 1.56, *p* = 0.12). There was also a near reliable interaction between *training-session* and *condition* (*β* = 0.10, SE = 0.05, *z* = 1.92, *p* = 0.054). Inspecting the graphs, this seems to reflect the fact that the difference between conditions emerges only in the second half of training. As a follow up, we explored whether the effect of variability changed from the first half of the experiment (Sessions 1 to 5) to the second half of the experiment (Sessions 6 to 10) in an identical statistical model where *training-session* was replaced by the binary factor *test-half* (Sessions 1 to 5 versus 6 to 10). In this model, there was a reliable effect of *test-half* (*β* = 1.05, SE = 0.14, *z* = 7.51, *p* < 0.001), which broke down to show no reliable effect of variability in the first five sessions (*β* = 0.08, SE = 0.24, *z* = 0.32, *p* = 0.75) but a reliable effect of variability (benefitting low-variability) in last five sessions (*β* = 0.56, SE = 0.23, *z* = 2.44, *p* = 0.015).

Although, we were not able to include the interactions with talker in the original model (with *training-session* as a continuous variable), in the follow up model (with *test-half* replacing *training-session*) the interaction between *test-half* and *condition* was qualified by a reliable effect of talker (*talker* by *test-half* by *condition*: *β* = 0.73, SE = 0.34, *z* = 2.16, *p* = 0.031). Breaking this down, there was a reliable effect of variability only for *female 2* (the more intelligible talker) in the second half of training (**Female 1, first half:** *β* = −0.05, SE = 0.31, *z* = −0.16, *p* = 0.88; **Female 1, second half:** *β* = 0.09, SE = 0.33, *z* = 0.27, *p* = 0.79; **Female 2, first half**: *β* = 0.22, SE = 0.29, *z* = 0.756, *p* = 0.45; **Female 2, second half:** *β* = 1.09, SE = 0.29, *z* = 3.83, *p* < 0.001).

#### Age group comparisons

Adults in the low-variability condition hit ceiling by the final training sessions, making statistical comparisons with children inappropriate. Restricting analysis to the high-variability conditions (and returning to a model with *training-session* as a continuous variable): there was a main effect of *age-group* (*β* = −1.01, SE = 0.22, *z* = −4.64, *p* < 0.001) reflecting the overall higher performance of adults, however, critically, there was no reliable interaction between *age-group* and *training-session* (*β* = −0.05, SE = 0.05, *z* = −1.02, *p* = 0.31). Thus, there was no reliable evidence of faster learning in children than in adults.

#### Summary of training data

All participants improved overall in the training task over time. For adults, there was a benefit of *low-variability*; observable throughout training and an interaction with session suggesting that this benefit increased throughout training. For children, there was no overall benefit of low-variability; however, there was marginal evidence of greater improvement in the *low-* rather than *high-variability* training. This seems to emerge in the second half of training and to be only true for the more intelligible speaker. In contrast to Giannakopoulou, Uther & Ylinen (2013a), there was no evidence of a “plasticity” benefit whereby children showed larger benefits of training. Instead, in the low-variability condition, adults (but not children) were at ceiling by the end of training, and in the high-variability condition there was no reliable difference in the improvement shown by adults and children.

### English Introduction

The English introduction task was included primarily to check whether participants knew the meanings of any of the English words prior to the experiment. However, we were also able to use this test to explore whether knowledge of word meanings improved following training. Accuracy in selecting the correct word (from a choice of three foils) on the first attempt was coded as 1/0. Table 4 shows the percentage of correct trials for adults and children in each condition pre- and post-training. Both groups appear to improve with training, although adults outperform children and are close to ceiling (above 80% correct, even at pre-test, with many participants having perfect scores). Given ceiling effects, in the adult data, we restricted our statistical analyses to the data collected from children. A logistic mixed effects model was run over the child data predicting their accuracy (1/0) with fixed factors of *test-session* (pre-test, post-test), *condition* and *test-session* by *condition*, as well as the control factor of *talker* and all of the interactions. This revealed a reliable main effect of *test-session* (*β* = 3.19, SE = 0.26, *z* = −12.23, *p* < 0.001) and a marginal interaction between *test-session* and *condition* indicating perhaps more learning in the low-variability condition (*β* = −0.95, SE = 0.51, *z* = −1.87, *p* = 0.061).

**Table 4:** Performance in the English introduction task: adult and children’s knowledge of word meanings at pre- and post-test (standard error in parentheses).

		Pre-test	Post-test
Adults	High-variability condition	81% (2%)	98% (2%)
	Low-variability condition	82% (2%)	100% (2%)
Children	High-variability condition	50% (3%)	88% (3%)
	Low-variability condition	47% (2%)	92% (2%)

In summary, both adults and children showed a pattern of improved knowledge of the word meanings from pre- to post-test with no differences between the high-variability and low-variability conditions, although it was only possible to statistically verify these patterns for children due to ceiling effects in adults.

### Primed auditory lexical decision

**Figure 4:**
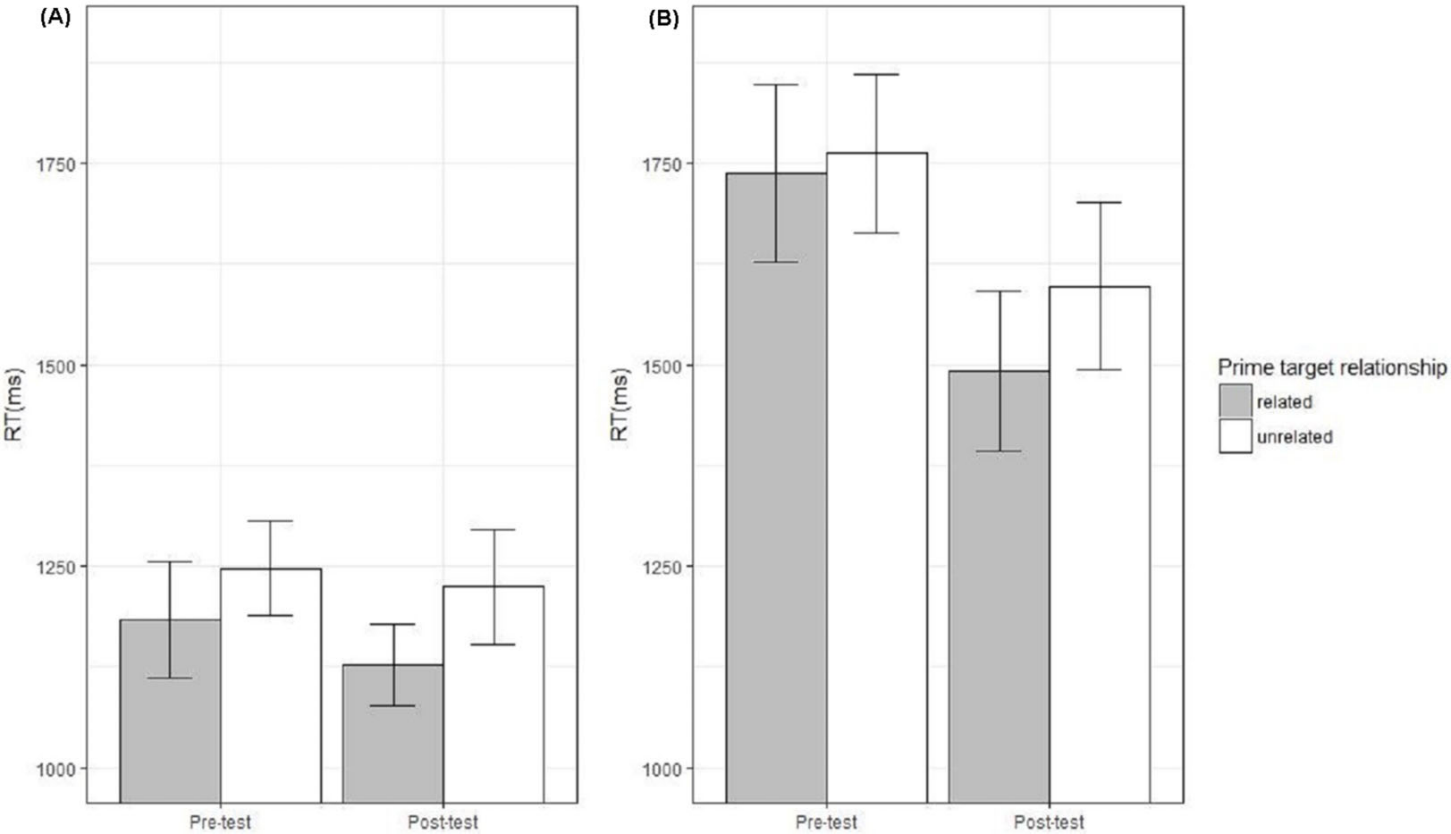
(A) Adult and (B) child performance in the primed auditory lexical decision task collapsing across condition. Mean RTs for Greek target words with related and un-related English primes pre- and post-test. Error bars show standard error (note: for all of the plots within this paper, means are corrected to control for imbalance in counterbalancing of talkers).

Trials with nonword targets were excluded. We conducted separate analyses for trials with Greek and English primes (targets were always Greek words). The Greek-primes analyses allowed us to determine whether standard semantic priming occurs within the native language, and thus served as a check on our experimental set up. (Note that, in addition to the means supplied within the text and in Fig. 4, a full break down of means by condition can be seen in the R script at http://rpubs.com/ewonnacott/247911).

For the Greek-primes analyses, trials on which targets were incorrectly identified as nonwords were removed (adults = 5%; children = 10%), as were trials with RTs <200 ms or >2.5 SD above the mean for each participant in each test-session (i.e., a further 3% of data for adults, 3% for children). The remaining data were analyzed in a linear mixed effects model predicting RT with fixed factors of *prime-target relationship* (related, unrelated), *test-session* (pre-, post-test) and the interaction between them.

For English-primes analyses, we analyzed both RTs (for children and adults), and accuracy (children only). For the RT analyses, data were filtered as described above (incorrect trials: adult = 6%, children = 16%; additional data removed due to <200 ms or >2.5 SD filter: adults = 4%, children = 4%). The remaining data were analyzed in a linear mixed effects model predicting RT with fixed factors of *prime-target relationship* (related, unrelated), *test-session* (pre-, post-test) and *condition* (high-variability, low-variability), the control variable *talker* and all of the interactions between these factors. For the accuracy analyses, all English-prime trials with real word targets were included and the data were analyzed using a logistic linear mixed effects model predicting accuracy of response (i.e., whether the target was correctly identified as a real word—coded as 1/0) with the same predictors as the RT data.

#### Adults

For *Greek primes,* there was a significant main effect of *prime-target relationship* (*β* = 101.33, SE = 13.52, *t* = 7.40, *p* < 0.001, related = 1,014 ms, unrelated = 1,114 ms), reflecting a standard priming effect for semantically related words within the native language. There was no overall effect of *test-session* (*β* = 37.90, SE = 39.45, *t* = 0.95, *p* = 0.35) and no interaction between *test-session* and *prime-target relationship* (*β* = 17.71, SE = 26.41, *t* = 0.67, *p* = 0.507).

For *English primes*, there was a significant main effect of *prime-target relationship* (*β* = 83.62, SE = 20.13, *t* = 4.04, *p* < 0.001; related = 1,153 ms, unrelated = 1,238 ms), reflecting semantic priming across the two languages. There was no overall effect of *test-session* (*β* = 42.18, SE = 48.64, *t* = 0.82, *p* = 0.41), suggesting no change in RTs in the post-test. Of critical interest is whether there was an interaction between *test-session* and *prime-target relationship*, since this could indicate an effect of training on priming. No such effect was found (*β* = −37.26, SE = 41.76, *t* = −0.85, *p* = 0.40) and there was no three-way interaction between *test-session, prime-target relationship* and *condition* (*β* = 95.90, SE = 83.81, *t* = 1.10, *p* = 0.28). Thus, there was no evidence that across language semantic priming was affected by the training (see Fig. 4).

#### Children

For *Greek primes* there was a significant main effect of *prime-target relationship* (*β* = 72.34, SE = 29.21, *t* = 2.47, *p* = 0.017, related = 1,487 ms, unrelated = 1,549 ms), reflecting a standard priming effect for related words within the native language. There was a marginal overall effect of *test-session* (*β* = 136.08, SE = 69.21, *t* = 1.95, *p* = 0.057), reflecting a slight reduction in RT length from pre (1,563 ms) to post (1,469 ms) test for children. There was no interaction between *test-session* and *prime-target-relationship* (*β* = −25.25, SE = 67.97, *t* = −0.37, *p* = 0.71).

For *English primes* (RT data) there was a marginal main effect of *prime-target relationship* (*β* = 65.17, SE = 33.39, *t* = 1.95, *p* = 0.058, related = 1,620 ms, unrelated = 1,664 ms), reflecting across language semantic priming. There was an overall effect of *test-session* (*β* = 181.88, SE = 74.46, *t* = 2.34, *p* = 0.023) indicating decreasing RTs from pre (1,704 ms) to post (1,580 ms) test. However, there was no interaction between *test-session* and *prime-target relationship* (*β* = −52.67, SE = 66.60, *t* = −0.79, *p* = 0.43) and no three-way interaction between *test-session, prime-target relationship* and *condition* (*β* = −7.55, SE = 133.81, *t* = −0.06, *p* = 0.96), suggesting that across language priming was not affected by the training (see Fig. 4).

Given the large amount of data excluded from the previous analyses of *English primes* (i.e., 16% of words inaccurately identified as nonwords), we also analyzed the accuracy data for children. Similar results were obtained. There was a significant main effect of *prime-target relationship* (*β* = −0.71, SE = 0.13, *z* = −5.60, *p* < 0.001, related: 87%, unrelated 81%), reflecting across language semantic priming. However, there was no interaction between *test-session* and *target-relationship* (*β* = 0.26, SE = 0.23, *z* = 1.12, *p* = 0.26), and no three-way interaction between *test-session, semantic priming* and *condition* (*β* = 0.01, SE = 0.40, *z* = 0.03, *p* = 0.98).

#### Summary of primed auditory lexical decision data

Analyses of Greek-prime trials established that both adults and children showed standard semantic priming effects within their native language (i.e., shorter RTs for targets preceded by related compared to unrelated primes), which held steady across the two test-sessions. Analyses of English-prime trials demonstrated that both adults and children showed evidence of across language priming (for adults, reliably shorter RTs, for children marginally shorter RTs and reliably more accurate responses). However, for both age groups, there was no evidence of an increase in the degree of semantic priming following training.

### Three-interval oddity discrimination test

**Figure 5:**
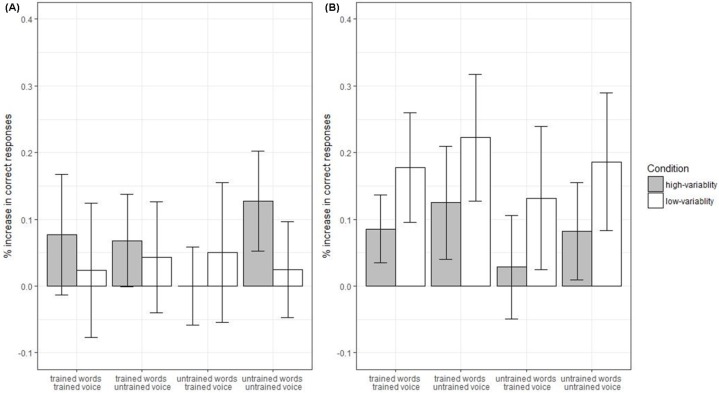
(A) Adult and (B) child discrimination data. Mean increase in percent correct responses from pre- to post-test (error bars show standard error). Note: for all of the plots within this paper, means are corrected to control for imbalance in counterbalancing of talkers.

We first ran separate logistic mixed effects models for each age-group, predicting whether a participant gave a correct response (i.e., picked the correct word as different out of a choice of three, coded as 1/0) on each trial. The fixed factors were *test-session* (pre-test, post-test), *voice-novelty* (trained voice, untrained voice), *word-novelty* (trained word, untrained nonword), *condition* (high-variability, low-variability) and all the interactions between these experimental factors. The factor *talker* and all of the interactions between *talker* and the experimental factors were included as control factors. We also included the continuous variable *mean-replay-usage* as an additional control factor. We ran a further model over the combined data from children and adults which included the same factors as before as well as the fixed effects of *age-group* (adult, child) and all of the interactions with this factor. This model was specifically inspected to look for the effects of *age-group*. (Note that, in addition to the means supplied within the text and the difference scores in Fig. 5, a full break down of means at pre and post-test by condition can be seen in the R script at http://rpubs.com/ewonnacott/247911).

#### Adults

There was no reliable main effect of *word-novelty* (*β* = −0.15, SE = 0.12, *z* = −1.23, *p* = 0.22), suggesting no difference in discrimination for real English words compared to nonwords. There was a reliable effect of *test-session,* indicating an effect of training (*β* = −0.52, SE = 0.15, *z* = −3.44, *p* = 0.001). This was qualified by a reliable interaction with talker (*β* = −0.59, SE = 0.23, *z* = −2.53, *p* = 0.01), which broke down to show that though there was numerical improvement from pre- to post-test for both talkers, this was only reliable for the more intelligible talker (Female 2: *β* = −0.82, SE = 0.23, *z* = −3.61, *p* < 0.001; 87% → 93%; Female 1, *β* = −0.23, SE = 0.15, *z* = −1.51, *p* = 0.13: 66% → 70%) (note that there were no significant higher level interactions involving *talker*—see Table S3).

Of critical interest is how participants’ improvement from pre- to post-training was affected by input condition and the novelty manipulations. This is depicted in terms of difference scores in Fig. 5. There were no reliable interactions of *test-session* by *word-novelty* (*β* = 0.07, SE = 0.25, *z* = 0.29 *p* = 0.77), *test-session* by *voice-novelty* (*β* = 0.19, SE = 0.25, *z* = 0.76, *p* = 0.45), or *test-session* by *word-novelty* by *voice-novelty* (*β* = −0.82, SE = 0.50, *z* = −1.67, *p* = 0.10). Contrary to predictions, adults did not show reliably greater improvement in the *high-variability* than *low-variability* conditions (*test-session* by *condition*, *β* = 0.07, SE = 0.29, *z* = 0.22, *p* = 0.82). There was also no *word-novelty* by *test-session* by *condition* interaction (*β* = 0.23, SE = 0.46, *z* = 0.50, *p* = 0.62). However, there was a reliable three-way *voice-novelty* by *test-session* by *condition* interaction (*β* = −0.91, SE = 0.46, *z* = −1.99, *p* = 0.046), which was qualified by a four-way *voice-novelty* by *word-novelty* by *test-session* by *condition* (*β* = 1.94, SE = 0.92, *z* = 2.11, *p* = 0.035). Breaking down the four-way interaction, *voice-novelty* by *test-session* by *condition* interaction was not reliable for trained words (*β* = 0.06, SE = 0.62, *z* = 0.10, *p* = 0.93) but was for untrained words (*β* = −1.88, SE = 0.67, *z* = −2.79, *p* = 0.005). Breaking down the three-way interaction for untrained words, there was a marginal *condition* by *session* interaction for the untrained voice which went in the predicted direction (i.e., more benefit of high-variability for the untrained voice; *β* = 0.89, SE = 0.50, *z* = 1.76, *p* = 0.078) but also a marginal interaction in the opposite direction for the trained voice (*β* = −0.99, SE = 0.52, *z* = −1.91, *p* = 0.056). In other words, the interaction rests both on a trend towards a greater benefit of *high-variability* input compared with *low-variability* input for untrained words-untrained voice items (which is predicted since novelty should aid generalization) and a trend towards a greater benefit of *low-variability* for *untrained words-trained voice* items (which is not predicted).

#### Children

There was no reliable main effect of *word-novelty* (*β* = −0.03, SE = 0.07, *z* = −0.38, *p* = 0.71), suggesting no difference in discrimination for real English words as opposed to nonwords. There was a reliable effect of *test-session*, indicating an effect of training (*β* = −0.67, SE = 0.10, *z* = −6.77, *p* < 0.001, pre-test = 62%, post-test = 74%). Again improvement from pre- to post-test is of critical interest and the relevant difference scores are shown in Fig. 5. There was a marginal interaction between *word-novelty* and *test-session* (*β* = −0.25, SE = 0.15, *z* = −1.73, *p* = 0.084), reflecting a slightly larger improvement for the trained words (60% → 75%) than the untrained nonwords (63% → 74%). In contrast to adults, there was a reliable interaction between *test-session* and *condition* (*β* = −0.49, SE = 0.20, *z* = −2.48, *p* = 0.013), with children showing a *reversed* effect to that predicted—i.e., reliably greater improvement in the *low-variability* condition (18%) than the *high-variability* condition (8%). However, participants in the low-variability condition were (by chance) lower at pre-test (*β* = −0.49, SE = 0.18, *z* = −2.68, *p* = 0.007; low-variability = 56%, high-variability = 66%) and in fact have not overtaken by post-test (*β* = 0.00, SE = 0.19, *z* = −0.02, *p* = 0.99; low-variability = 74%, high-variability = 74%). The *test-session* by *condition* interaction was not qualified by an interaction with *word-novelty* (*β* = 0.07, SE = 0.29, *z* = 0.25, *p* = 0.80) *voice-novelty* (*β* = −0.02, SE = 0.31, *z* = −0.06, *p* = 0.95) or *word-novelty* by *voice-novelty* (*β* = 0.09, SE = 0.29, *z* = 0.29, *p* = 0.77).

#### Age-group comparison

There was a main effect of *age-group*, reflecting overall higher performance in adults than children across pre- and post-test (*β* = −0.81, SE = 0.13, *z* = −6.43, *p* < 0.001, adults = 79%, children = 68%). Critically, although numerically children improved more from pre- to post-test (see Fig. 5) there was no reliable interaction between *age-group* and *test-session* (*β* = −0.17, SE = 0.17, *z* = −1.02, *p* = 0.31). The *age-group* by *test-session* interaction was not involved in any reliable higher level interactions with any combination of *condition, word-novelty* or *voice-novelty* (*p*’s > 0.1) although there was a near reliable five-way interaction of *condition* by *word-novelty* by *voice-novelty* by *age-group* by *test-session* (*β* = −2.00, SE = 1.04, *z* = −1.92, *p* = 0.055), reflecting the different effects of these factors in the adult and child models reported above.

#### Summary of discrimination data

Adult participants improved in their discrimination performance from pre- to post-test, suggesting an effect of training. Although, numerically, adults showed greater improvement in the high- compared to the low-variability condition, the difference was not reliable (or near reliable). There was some tentative evidence of an interaction between variability and novelty, with the greatest effect of variability evident for maximally novel items. However, the key interaction rested both on a predicted benefit of high-variability training for items with untrained words and voices *and* an unpredicted benefit of low-variability training for items with untrained words and the trained voice, making it difficult to interpret. Child participants also showed a benefit of training in improvement from pre- to post-test. In contrast to adults, children *did* show an overall effect of variability, although this was in the opposite direction to that predicted, with greater improvement in the *low-* compared to *high-variability* condition, with no evidence that this was affected by the *novelty* of either word or voice used in the test items or by the *talker* used as the new or old voice. However, this interaction was driven by (chance) differences between conditions at pre-test, rather than differences at post-test, and should thus be treated with some caution. In contrast to the previous study by Giannakopoulou, Uther & Ylinen (2013a), children did not show reliably greater improvement from pre- to post-tests than adults, i.e., we did not replicate the “plasticity” effect seen in that study.

## General Discussion

The current study compared the effects of talker variability in phonetic training in eight-year-olds and adults. Native Greek learners of English were trained to discriminate the nonnative English /i/-/ɪ/ contrast in ten training sessions using a picture identification task in which they heard a target word (e.g., *sheep*) and chose between pictures of the target (*sheep*) and its minimal pair counterpart (*ship*). Critically, half of the participants heard a single talker during training (low-variability input) whilst the other half heard four talkers (high-variability input), with items and frequencies matched across conditions. Training performance was recorded and we administered pre- and post-tests, including a three-interval oddity discrimination test, which tapped participants’ ability to discriminate the /i/-/ɪ/ contrast, and tests tapping knowledge of the trained vocabulary. We predicted greater increases in performance following *high-variability* training, given the literature on benefits of high-variability training in both phonetic learning (Bradlow & Bent, 2008; Clopper & Pisoni, 2004; Lively, Logan & Pisoni, 1993) and vocabulary learning (Barcroft & Sommers, 2005, 2014; Sommers & Barcroft, 2007, 2011). Data did not support this prediction. We also expected that children would show greater increases in performance than adults, at least in the training and discrimination tasks, given the findings of Giannakopoulou, Uther & Ylinen (2013a). Again, this was not seen in the data. In this discussion, we first consider the findings from each task, focusing on the contrast between *high-* and *low-variability* input. We then turn to age-related differences, considering why we do not see the same benefit for child learners seen in previous studies, and the implications for theories of plasticity and maturation.

### Training task

All groups showed improvement across training sessions. Both adults and children showed consistently stronger performance following low- rather than high-variability input. However, for children a benefit for low-variability training only emerged in the second half of training, and only with the more intelligible speaker.

From the perspective of phonetic discrimination, greater performance following low-variability training is perhaps unsurprising. First, repeated exposure to the same items produced by the same talker potentially allows participants to attune to idiosyncratic cues associated with that talker (Clopper & Pisoni, 2004). In addition, the fact that our talkers varied on a trial by trial basis meant that trial by trial adaptation to talker was required in the high-variability condition, possibly imposing a burden on learners in that condition (see Martin et al., 1989; Nusbaum & Morin, 1992 for evidence that multitalker stimuli are difficult even for L1 processing). Given this, it is perhaps surprising that children did *not* show a reliable benefit of low-variability until the second half of training since we might actually expect that their lower working memory capacity would increase the benefit for low-variability (Nusbaum & Morin, 1992; see below for further discussion of this in relation to the discrimination data). However, one confounding factor here is the evidence from the pre-training discrimination test which indicates that the low-variability children started out, by chance, somewhat lower in their ability to discriminate these contrasts, making it hard to evaluate differences in the first half of training.

Given that our task can also be viewed as a word-learning task, it is worth considering how this result fits with that of Rost & McMurray (2009), who found that 14-month-olds, who are developing their knowledge of L1 phonetic contrasts, only learn two minimal pair object labels when those words were spoken by multiple talkers, *not* when they were spoken by a single talker. This was despite the fact that test items did not probe generalization, testing with a voice familiar from exposure. Similarly, Barcroft & Sommers (2005) found benefits of multiple-talker training for adults learning novel words from a foreign language, and their tests included L2 to L1 translation where the test items used talkers familiar from training. One possibility is that in our training task, any potential benefit of variability may have been attenuated by the necessity of continuously adapting to a new speaker on a trial by trial basis, as discussed above.

### Three-interval oddity discrimination test

In the three-interval oddity discrimination test, participants identified the odd man out from a choice of three words (e.g., *sheep, sheep, ship*). We were interested in the extent of improvement from pre- to post-test, and whether this was affected by training condition and novelty (of either words or talkers). If high-variability is specifically useful in supporting generalization (as argued in the phonetic training literature), we predict that high-variability training should benefit generalization items. Results from adult participants were, to some extent, in line with this prediction, with numerically greater improvement in the high-variability condition, however, this difference was not statistically reliable. The lack of a reliable difference between conditions may be due to the overall high performance of adult participants in this test. There was some evidence of an interaction between novelty and the benefit of variability. However, although a greater benefit of high-variability for more novel items is predicted (i.e., because it allows the formation of generalized representations that include only phonetically relevant cues and exclude irrelevant talker identity cues), the interaction relied in part on a benefit for the *low-*variability group for novel items with the familiar talker, which was not predicted. This makes the result difficult to interpret. It is notable that the strongest evidence for the benefit of high-variability training has come from studies using identity tests (Lively, Logan & Pisoni, 1993; Sadakata & McQueen, 2013). This type of test was *not* possible in the current context, where we did not use orthography, but if high-variability is specifically useful in the formation of category level representations, it may be that an identity test is more useful for testing this type of learning.

As for children, surprisingly, there was reliably greater improvement following low- rather than high-variability training. This held regardless of the novelty of test items. One concern in interpreting this result is that our low-variability group (by chance) began with lower scores at pre-test. Our analyses focus on changes from pre- to post-test (i.e., we examine interactions with test session); however, it is possible that the pre-test difference could be biasing since the high-variability group have less space for improvement (although it is worth noting that our statistical analyses were not done over proportions, but using logistic regression via mixed models which should be less susceptible to this problem). One interpretation of this result is that, for children, the four speaker input may contain too many varying cues which serve to obfuscate the critical cues needed for distinguishing the /i/-/ɪ/ contrast. This is in line with the Active Control Hypothesis (Magnuson & Nusbaum, 2007), which views speech perception as an active processes of balancing bottom-up and top-down expectations and constraints. According to this hypothesis, continuous adaption to a new speaker may usurp working memory capacity. This is supported by experimental evidence suggesting that L1 speech recognition may be slowed when listeners are placed under working memory load (remembering visually presented numbers), but only if there were multiple talkers (Nusbaum & Morin, 1992). Since children are known to have lesser phonological working memory than adults, the burden placed by high-variability input may leave them relatively fewer resources for phonetic learning. However, replication with samples which are deliberately matched at pre-test is important (cf., Antoniou & Wong, 2016) since this benefit of low-variability is unexpected, particularly for new items where it is difficult to see how more limited exposure could actually benefit generalization.

### English introduction test

In the English introduction task, participants matched the meaning of a Greek word to its English counterpart given a choice of four words. One purpose of this task, which included feedback, was to ensure that all participants began the experiment with knowledge of the meanings of the words before beginning training. However, it was administered pre- and post-test and thus also provides a measure of participants improved knowledge of the words. Performance even at pre-test was very high, and all adults were at ceiling at post-test making analysis of their data inappropriate. However, children’s data were not at ceiling and were analyzed. This revealed an improved knowledge of word meanings from pre- to post-test, but no effect of variability condition. This contrasts with the results of Barcroft & Sommers (2005) who found a benefit of multiple talker input for adult vocabulary learning. Given the lack of appropriate comparison data from adults in the current study (i.e., due to the ceiling effects), further work is needed to establish whether the difference we see here is due to the children’s age or to one or more of the many differences in our paradigm such as: (i) the fact that our learners are not novices but begin with some knowledge of the words (ii) the fact that we have multiple (10) learning sessions (iii) the focus on discriminating a nonnative speech-sound during training (i.e., via the minimal pairs task) rather than simple exposure to objects and phonological labels as generally occurs in their tasks.

### Primed auditory lexical decision test of semantic priming

The aim of the auditory lexical decision task was to see if semantic representations were affected by the training, and whether the greater “robustness” of lexical representations reported for high-variability training in previous work would extend to semantic representations. Specifically, we looked for increases in semantic priming from pre- to post-test and tested whether this was greater in the high-variability condition. Analyses revealed that, while both adults and children showed reliable across language semantic priming (revealed in faster RT’s for adults and greater accuracy for children), this was present even before training and there was no evidence of an increase in priming after the training in either condition for either group. This was contrary to our expectation that repeated exposure to the words with their picture depictions would *increase* the robustness of those representations and thus increase semantic priming. This does not appear to occur, or at least not sufficiently to be detected by this test. Given that there is no evidence of changes to semantic representations in *either* condition, it is not possible to interpret the lack of evidence for a difference in talker variability for this measure. Future work could consider whether different types of training (e.g., using multiple pictures to represent each word during training, or presenting words in richer contexts such as meaningful sentences) are more effective in this respect.

### Maturational differences

In the current study adults generally outperformed children both at pre- and post-test. This result is not surprising from the perspective of word learning, where adults typically outperform children in recognition and recall of new words (Henderson et al., 2013). However, Giannakopoulou, Uther & Ylinen (2013a) found that children of a similar age to those in the current study, showed greater learning of the /i/-/ɪ/ distinction than adults, as shown both in training (where adults initially out-performed children but were overtaken by the final training session) and in the three-interval oddity discrimination test (where children showed reliably greater improvements from pre- to post-test than adult participants). In contrast, in our training data children did *not* overtake adults, instead, it was adults who reached ceiling in the low-variability, while in the high-variability condition (most similar to the training in Giannakopoulou, Uther & Ylinen, 2013a) our analyses found no evidence that children improve more from pre- to post-test than do adults. Given that we focus on the same phonetic contrasts, and use similar methods and tests, the reasons for these differences are unclear. First, we acknowledge the importance of not over-interpreting a null effect—we have no evidence of an age effect, rather than evidence of a *lack* of an age effect. We may simply not have sufficient power. The different findings could also be due to differences in our participant samples. We note that the average age of the children in the current study is slightly higher than in the previous study (current study: 8;9 years; Giannakopoulou, Uther & Ylinen: 7;11 years). There are also differences in the extent of participants’ previous English experience, which was greater in the current study for both age groups (current study: children average of 1.96 years, adult average of 10.98 years; previous study: children average of 1.4 years; adult average of 8.7 years). Note that previous English experience is a confounding factor with age in *both* the current study and Giannakopoulou, Uther & Ylinen (2013a)—adults have substantially more experience in both cases—however, the greater extent of experience for both groups in the current study could potentially limit the opportunity to see maturational differences. Speaking against this, we note that at pre-test our adults and children performed quite similarly in the three-interval oddity discrimination tests to those in the previous study; our adults: 76%, our children: 62%; Giannakopoulou, Uther & Ylinen (2013a): adults 92%, children 76%.

Another possibility is that there is a different role for age in the two studies, due to differences in the training and testing tasks. One key difference in training is the use of picture rather than orthographic stimuli. As noted in the introduction, for this specific contrast, English orthography provides an analogue cue to the perceptual length difference between the two vowels—the shorter vowel is generally transcribed with a single letter (*i*) and the longer vowel with a digraph (*ee*/*ea*). Recall that Giannakopoulou, Uther & Ylinen (2013a) found that children’s greater improvement over adults was particularly marked in the condition where natural auditory stimuli were used in training, compared with a condition where the stimuli had been modified to remove length cues. One possibility is that children may make particular usage of the match between the length of the auditory and visual stimuli, leading to their lesser success in the current experiment where this cue was not provided. An additional benefit of orthography is that it provides consistent cues *across trials* as to vowel category—i.e., there are letters/pairs of letters which occur across different items with the same vowel (i.e., *i in chip*, *bid* and *lick*, versus *ea* in *cheap*, *bead* and *leak*). Thus when orthography is present, learners can focus on more general mappings between the orthographic units and the vowels, potentially ignoring the rest of the lexical item, whereas in the current study they have to learn how each vowel maps to each picture on an idiosyncratic basis. It is possible that this is particularly challenging for children compared with adults. However, it is worth recalling that there are other phonetic training studies that have also not found a benefit for younger learners, and these *did* provide consistent cross-trial cues [e.g., Wang & Kuhl (2003), asked participants to choose consistent symbols for each of the four tones; Heeren & Schouten (2008, 2010), asked them to match to “long t”]. However, as discussed above, other factors may contribute to the lack of benefit to child learners in these cases.

Furthermore, research is needed to pull apart the reasons that a benefit in children is seen in Giannakopoulou, Uther & Ylinen (2013a) but not in the current study, and more broadly to establish which factors are important in child and adult learning. However, it is clear that the current results do not support a clear story in which children’s greater plasticity leads them to benefit more from phonetic training and it seems likely that there are interactions with task complexity.

## Conclusions and Future Directions

The current experiment adds to the literature demonstrating that L2 learners can improve their discrimination of a phonetic contrast via computerized phonetic training. In particular, we add to the handful of studies demonstrating that is true for child L2 learners.

In contrast to previous literature, although performance of both adults and children improved across training, and discrimination scores improved from pre- to post-training, we did not find evidence of greater improvements for learners trained on input produced by multiple talkers compared with a single talker. Instead, both age groups showed benefits of hearing a *single* talker within the training task and there was some evidence that children showed this same benefit in the discrimination test. We also did not see any benefit of high-variability in terms of word learning, either in the semantic priming test or the basic vocabulary test. In the above discussion, we have considered possible explanations for the discrepancy between these results and the previous literature showing a high-variability benefit. There are various differences between both the training and testing tasks which could account for the differences and future work must tease these apart. In particular, in the current work, since we were not using orthography, we did not include a pre- and post-test “identity” test, but this makes the result harder to compare, and we are developing methods for testing this in future work. Future work will also address whether “blocking” the input by speaker in the high-variability condition is necessary in order to see the benefits of this type of exposure. We also intend to include a word production test in future work. This will both serve to explore the extent to which comprehension training is generalized to production and also provide a vocabulary test more akin to that used in the relevant word learning literature.

Our results also do not support the findings of greater plasticity in child learning found by Giannakopoulou, Uther & Ylinen (2013a). Additional work is necessary to pull apart the benefits of directly representing phonemes during training using orthography (or some other categorical representation system) and whether this is particularly important for child learners.

A final point of interest in our data is that, despite the fact that we used semantic representations (pictures) when training the words, there was no evidence that training increased the robustness of the semantic representations, at least as captured by the semantic priming task. This raises the important question of the extent to which the type of minimal pairs training employed here, and elsewhere, actually changes learners’ L2 lexical representations. We consider this to be a key question for future research.

## Supplemental Information

10.7717/peerj.3209/supp-1 Supplemental Information 1 Table S1. Minimal Pair Stimuli.

10.7717/peerj.3209/supp-2 Supplemental Information 2 Table S2. Stimuli for the Primed Lexical Decision Task.

10.7717/peerj.3209/supp-3 Supplemental Information 3 Table S3. Analyses with the Control Variable “Talker”.

## Additional Information and Declarations

### Data Availability

The following information was supplied regarding data availability: The analyses scripts and output can be viewed here: http://rpubs.com/ewonnacott/247911. For data files and scripts, see the Open Science Framework: https://osf.io/8anzk/. For a full breakdown of means by condition, please refer to the accompanying R script, available at: http://rpubs.com/ewonnacott/247911. For a full breakdown of means at pre- and post-test by condition, please refer to the accompanying R script, available at: http://rpubs.com/ewonnacott/247911.
