# Supplementary material for: High or low? Comparing high and low-variability phonetic training in adult and child second language learners
Source: PeerJ. 2017 May 30;5:e3209. doi: 10.7717/peerj.3209 (PMC5452958; doi:10.7717/peerj.3209)
Supplement: Supplemental Information 1 [file peerj-05-3209-s001.pdf]

S1 Table. Minimal Pair Stimuli

Real Word Minimal Pairs		Non-Word Minimal Pairs	
Bead	Bid	Cheed	Chid
Bean	Bin	Dreest	Drist
Cheap	Chip	Dreet	Drit
Cheek	Chick	Feem	Fim
Deep	Dip	Freen	Frin
Feast	Fist	Freep	Frip
Gene	Gin	Freet	Frit
Heat	Hit	Gleep	Glip
Lead	Lid	Heen	Hin
Leap	Lip	Kreen	Krin
Leave	Live	Leel	Lil
Leek	Lick	Meep	Mip
Meal	Mill	Meev	Miv
Peach	Pitch	Preel	Prill
Peel	Pill	Preep	Prip
Sheep	Ship	Reet	Rit
Sleep	Slip	Steen	Stin
Sleet	Slit	Steet	Stit
Teen	Tin	Yeed	Yid
Wheel	Will	Yeet	Yit
