# Supplementary material for: High or low? Comparing high and low-variability phonetic training in adult and child second language learners
Source: PeerJ. 2017 May 30;5:e3209. doi: 10.7717/peerj.3209 (PMC5452958; doi:10.7717/peerj.3209)
Supplement: Supplemental Information 2 [file peerj-05-3209-s002.pdf]

S2 Table. Stimuli for the Primed Lexical Decision Task.

**Prime-Type**	**Prime Word**	**Target Word**			
		Semantically-Related	***Semantically-Unrelated***	***Nonword 1***	***Nonword 2***
English	meal	γεύμα (meal)	γούβα (hole)	γόλμα	χόδι
	wheel	ρόδα (wheel)	δώρο (gift)	ρύδι	κλεμί
	pill	χάπι (pill)	πόδι (foot)	χίκι	μασός
	bean	φασόλι (bean)	μπαλόνι (balloon)	φακύλι	ήμεος
	tin	κονσέρβα (tin)	κονσέρτο (concert)	κονσίρμα	πιένο
	leave	αφήνω (leave)	ανάβω (light)	αφάνω	κάλμπα
	gene	γονίδιο (gene)	τζίπ (jeep)	τζέν	σκούτος
	lid	καπάκι (lid)	παπάκι (duck)	καπένι	πισούνι
	bid	δημοπρασία (bid)	φωτογραφία (photograph)	δημιπαρσία	ώκα
	fist	γροθιά (fist)	βραδιά (night)	γραλιά	φύσο
	cheap	φθηνός (cheap)	γλυκός (sweet)	φθελός	κεμισιά
	deep	βαθύς (deep)	γκρί (grey)	ντάκ	ημεκομόγιο
	heat	ζεσταίνω (heat)	κερνάω (treat)	ζεστάζω	θηκαυλός
	leek	πράσο (leek)	σκάλα (ladder)	πρόκο	φρούμο
	peach	ροδάκινο (peach)	τηλέφωνο (telephone)	ροδόλινο	μασιλόρι
	sheep	πρόβατο (sheep)	γόνατο (knee)	πράκατο	γραμόλι
	sleep	κοιμάμαι (sleep)	φοβάμαι (afraid)	κοιλάμι	τρέω
	slit	σχίζω (slit)	φτύνω (spit)	σχέκω	τρογαλώ
	chick	κοτοπουλάκι (chick)	τετραγωνάκι (square)	κοσολαράνι	περμιτώ
	lip	χείλος (lip)	κύβος (cube)	χούλες	ζωγριφάνω
Greek	χέρι (hand)	πόδι (foot)	βάλτος (swamp)	πίδε	βάλμος
	κλαδί (branch)	δέντρο (tree)	βαλίτσα (suitcase)	δάντρα	βαλίμα
	μαγιό (swimsuit)	πισίνα (pool)	γάλα (milk)	πιράνο	γόδα
	ήλιος (sun)	θάλασσα (sea)	γαρίδα (prawn)	καλίσα	γασόδα

πιάτο (dish)	ποτήρι (glass)	γέφυρα (bridge)	ποσήκι	γέμυρα
κάλτσα (sock)	παπούτσι (shoe)	σαλάτα (salad)	παρούμπι	δάλμυ
σκύλος (dog)	γάτα (cat)	δέμα (parcel)	γόκα	δούμα
πιρούνι (fork)	κουτάλι (spoon)	δάκρυ (tear)	κουμάσι	δίαμα
ώρα (time)	ρολόι (watch)	σοκολάτα (chocolate)	ροκέσι	σομοράτα
φύλο (leaf)	λουλούδι (flower)	ελάφι (deer)	μπουμπούλι	ελόσι
κερασιά (cherrytree)	κεράσι (cherryfruit)	ημερολόγιο (diary)	καρόβι	άδομο
γυαλιά (glasses)	καπέλο (hat)	φρούτο (fruit)	καπίλε	γυαριό
νερό (water)	μπουκάλι (bottle)	θησαυρός (treasure)	μπουκότι	νελό
ποτό (drink)	φαγητό (food)	έντομο (insect)	φαγάτι	πετέ
δάσος (forest)	φυτό (plant)	μαξιλάρι (pillow)	κυρό	δίσος
γρασίδι (grass)	κήπος (garden)	δίαιτα (diet)	κέπις	σανάτα
τρώω (eat)	μαγειρεύω (cook)	πληρώνω (pay)	μαγελίζω	βλαίμω
χορεύω (dance)	τραγουδώ (sing)	δέρνω (smack)	κομεύω	δάρνω
τρέχω (run)	περπατώ (walk)	αγοράζω (buy)	τράλω	ταλισεύω
χρωματίζω (paint)	ζωγραφίζω (draw)	ταξιδεύω (travel)	χρωμικίζω	ακοράνω
