# Supplementary material for: High or low? Comparing high and low-variability phonetic training in adult and child second language learners
Source: PeerJ. 2017 May 30;5:e3209. doi: 10.7717/peerj.3209 (PMC5452958; doi:10.7717/peerj.3209)
Supplement: Supplemental Information 3 [file peerj-05-3209-s003.pdf]

S3 Table. Analyses with the Control Variable "Talker".

Note that, as stated in the results section, in addition to the results reported in the main text, we also inspected each model reported as follows: (i) we looked at the main effect of the control variable *talker* (ii) wherever we had found a reliable main effect or interaction with the experimental factors, we looked to see this was qualified by a higher level interaction with *talker*. These analyses are not reported in the main text except in cases where there *was* an interaction and it broke down to show that the effect was only reliable for one of the two talkers.

Task	Age group	Co-Efficient	
Training	Adults	Talker *	beta = 2.11, SE = 0.28, z = 7.41, p < .001
		condition by talker	beta = 1.73, SE = 0.45, z = 3.82, p < .001
		training-session by talker	beta = 0.26, SE = 0.06, z = 4.18, p < .001
		condition by training-session by talker	beta = 0.26, SE = 0.11, z = 2.44, p = .015
		**female 1:** condition	beta = 0.72, SE = 0.29, z = 2.53, p = .012
		**female 1:** training-session	beta = 0.21, SE = 0.02, z = 10.41, p < .001
		**female 1:** condition by training-session	beta = 0.07, SE = 0.04, z = 1.98 p = .048
		**female 2:** condition	beta = 2.47, SE = 0.44, z = 5.60, p < .001
		**female 2:** training-session	beta = 0.49, SE = 0.06, z = 7.53, p < .001
		**female 2:** condition by training-session	beta = 0.33, SE = 0.11, z = 3.10, p = .002
Training	Children	talker	beta = 0.19, SE = 0.22, z = 0.85, p = 0.395
		test-half by talker	beta = 0.19, SE = 0.28, z = 0.67, p = .506
		condition by test-half by talker	beta = 0.73, SE = 0.34, z = 2.16, p = .031
		**female1, first half:** condition	beta = -0.05, SE = 0.31, z = -0.16, p = .875
		**female1, second half:** condition	beta = 0.09, SE = 0.33, z = 0.27, p = .786
		**female1, first half:** condition	beta = 0.22, SE = 0.29, z = 0.76, p = .45
		**female1, second half:** condition	beta = 1.09, SE = 0.29, z = 3.83, p < .001

Training (high variability)	Both	talker<br>age-group by talker<br>**female1:** age-group<br>**female2:** age-group	beta = 0.17, SE = 0.22, z = 0.80, p = .423<br>beta = -1.07, SE = 0.44, z = -2.46, p = .014<br>beta = -0.47, SE = 0.30, z = -1.53, p = .126<br>beta = -1.54, SE = 0.31, z = -4.94, p < .001
English Introduction	Children	talker<br>training-session by talker<br>training-session by condition by talker	beta =- 0.1, SE = 0.28, z = -0.35, p = .728<br>beta = 0.14, SE = 0.50, z = 0.28, p = .780<br>beta = -0.56, SE = 1.00, z = -0.56, p = .579
Primed Auditory Lexical Decision, English prime trials (RT)	Adults	talker<br>prime-target relationship by talker	beta = -47.84, SE = 57.93, t = -0.78, p = .438<br>beta = -71.58, SE = 40.29, t = -1.72, p = .09
Primed Auditory Lexical Decision, English prime trials (RT)	Children	talker<br>test-session: talker<br>prime-target relationship by talker	beta = -70.54, SE = 97.11, t = -0.70, p = .489<br>beta = -245.95, SE = 149.07, t = -1.58, p = .12<br>beta = -8.20, SE = 66.88, t = -0.12, p = .903
Primed Auditory Lexical Decision, English prime trials (accuracy)	Children	talker<br>test-session: talker<br>prime-target relationship by talker	beta = -0.20, SE = 0.26, z = -0.75, p = .453<br>beta = 0.13, SE = 0.23, z = 0.58, p = .566<br>beta = 0.63, SE = 0.32, z = 1.95 p = .052
Three-Interval Oddity Discrimination Test.	Adults	talker<br>talker by test-session<br>**female1**: test-session<br>**female2**: test-session<br>voice-novelty by test-session by condition by talker<br>word-novelty by voice-novelty by test-session by condition by talker	beta = 1.68, SE = 0.13, z = 12.66, p < .001<br>beta = -0.59, SE = 0.23, z = -2.53, p = .011<br>beta = -0.23, SE = 0.15, z = -1.51, p = .13<br>beta = -0.82, SE = 0.23, z = -3.61, p < .001<br>beta = 0.37, SE = 0.92, z = 0.40, p = .69<br>beta = 1.89, SE = 1.84, z = 1.03, p = .305
Three-Interval Oddity Discrimination Test.	Children	talker<br>test-session by word-type by talker<br>test-session by condition by talker	beta = 0.80, SE = 0.07, z = 10.82, p < .001<br>beta = -0.17, SE = 0.30, z = -0.56, p = .578<br>beta = -0.36, SE = 0.31, z = -1.16, p = .246
Three-Interval Oddity Discrimination Test.	Both	talker<br>talker by age group<br>**female1:** age-group<br>**female2:** age-group	beta = 1.17, SE = 0.07, z = 17.28, p < .001<br>beta = -0.83, SE = 0.14, z = -5.97, p < .001<br>beta = -0.40, SE = 0.13, z = -3.02, p = .003<br>beta = -1.22, SE = 0.16, z = -7.88, p < .001

*In each case, a positive coefficient for *talker* reflects greater performance with ***female2***, which was the more intelligible talker across tasks.
